# P-152. Chitinase-3-like Protein 1 is Associated with Stunting and Neurodevelopmental Delay in Ugandan Children Who Are HIV Exposed But Uninfected

**DOI:** 10.1093/ofid/ofae631.357

**Published:** 2025-01-29

**Authors:** Elspeth MacBain, Andrea Conroy, Sophie Namasopo, Robert Opoka, Michael Hawkes

**Affiliations:** University of British Columbia, Vancouver, British Columbia, Canada; Indiana University School of Medicine, Indianapolis, Indiana; Mulago Hospital and Makerere University, Kampala, Kampala, Uganda; Jinja Regional Referral Hospital, Jinja, Jinja, Uganda; University of British Columbia, Vancouver, British Columbia, Canada

## Abstract

**Background:**

Children who are HIV exposed but uninfected (cHEU) are at risk for impaired linear growth and neurodevelopment, which evolving evidence suggests may be associated with elevated inflammatory biomarkers. Chitinase-3-like protein 1 (CHI3L1) is produced by activated neutrophils and has been linked to clinical manifestations of systemic inflammation in children living with HIV. We aimed to explore CHI3L1 as a potentially relevant marker for adverse growth and neurodevelopment outcomes experienced by cHEU.

**Methods:**

This was a prospective cohort study conducted at two pediatric HIV centres in Uganda (Jinja Regional Referral Hospital and Kambuga District Hospital). We enrolled children at birth, born to mothers living with HIV, diagnosed prior to the pregnancy or at the time of delivery. We excluded children who were subsequently found to be vertically infected (n=8), children who died before 18 month of age (n=3), as well as those lost-to-follow-up and those with missing CHI3L1 measurement. Neurodevelopmental ability (rank) was assigned based on the standardized score of Malawi Developmental Assessment Tool (MDAT) milestones achieved at 18 months of age. CHI3L1 levels were quantified by ELISA (R&D Duoset, Minneapolis, MN, USA).

**Results:**

We included 153 cHEU (53% female) born between March 2016 and June 2018. At 18 months of age, 42%, 0.7%, and 2.8%, were severely stunted, wasted, and underweight, respectively. Performance on the MDAT was similar to Malawian norms. The median CHI3L1 level was 30 µg/L (IQR 18-47). CHI3L1 levels were inversely correlated with weight-for-age (ρ= -0.22, p=0.0091) and height-for-age (ρ= -0.24, p=0.0039) z-scores, but not the weight-for-height or head circumference-for-age z-scores. CHI3L1 levels were higher in children with severe stunting (median 40 µg/L, IQR 26-86) than those without severe stunting (median 27 µg/L, IQR 16-39, p=0.0010). CHI3L1 was inversely correlated with the standardized MDAT scores (ρ= -0.29, 0.00023). Lower scores in the language and gross motor domains were associated with higher CHI3L1 whereas scores in the fine motor and social domains were not associated with CHI3L1.

**Conclusion:**

CHI3L1 was associated with severe stunting and neurodevelopmental delay in our cHEU cohort in Uganda.

**Disclosures:**

**All Authors**: No reported disclosures

